# CD31 regulates metabolic switch in Treg migration attenuates rheumatoid arthritis

**DOI:** 10.1002/ctm2.70441

**Published:** 2025-08-15

**Authors:** Meijun Liu, Wengqiong Huang, Xiaoli Chen, Zongzhen Meng, Jiawen Yang, Loiola Rodrigo Azevedo, Xiaojiao Zheng, Hao Shen, Wei Jia, Aiping Lyu, Kenneth CP Cheung

**Affiliations:** ^1^ Phenome Research Center Hong Kong Baptist University Hong Kong China; ^2^ School of Life Science Southern University of Science and Technology Shenzhen Guangdong Province China; ^3^ Oroxcell, Parc Biocitech Romainville France; ^4^ Center for Translational Medicine and Shanghai Key Laboratory of Diabetes Mellitus Shanghai Sixth People's Hospital Affiliated to Shanghai Jiao Tong University School of Medicine Shanghai China; ^5^ Department of Orthopaedics Shanghai Sixth People's Hospital Affiliated to Shanghai Jiao Tong University School of Medicine Shanghai China; ^6^ Department of Pharmacology and Pharmacy The University of Hong Kong Hong Kong China

**Keywords:** CD31 ITIMs, metabolic switch, PFKFB3, RNF111/OGT pathway, Tregs migration

## Abstract

CD31 (PECAM‐1) plays a critical role in T cell migration, whilst its immunoreceptor tyrosine inhibitory motifs (ITIMs), Y663 and Y686, are recognised for their roles in endothelial function, the precise mechanism in regulating immune cell remains elusive. Here, we demonstrate that CD31 is essential for Treg migration. Upon ITIM engagement, CD31 activates and interacts with the protein tyrosine phosphatase SHP2. In vivo, CD31 Y663F gene transfer recapitulates the wild‐type migration phenotype, driven by a metabolic switch to fructose utilisation under the regulation of the PFKFB3 gene. Conversely, the Y686F mutation impairs Tregs migration by disrupting both glycolysis and the switch to fructose metabolism, thus promoting the mitochondrial function via activation of the RNF111/OGT pathway. Our findings reveal a novel role for CD31 ITIMs in orchestrating a metabolic that is switch crucial for Treg migration. This understanding of CD31 polymorphisms and their impact on Treg migration offers potential therapeutic avenues for autoimmune diseases, particularly rheumatoid arthritis (RA).

**Key points:**

- CD31 Y663F‐mutant Tregs exhibit a glucose‐to‐fructose metabolic shift, characterised by reduced glucose uptake and enhanced fructose utilisation regulated by PFKFB3.
- CD31 Y686F mutation disrupts both glycolysis and fructose metabolism in Tregs, shifting energy production towards mitochondrial function via the RNF111/OGT pathway.
- These findings highlight a novel mechanism by which CD31 ITIMs control Treg migration, offering new therapeutic targets for autoimmune diseases such as RA.

## Graphical abstract

This study elucidates metabolic reprogramming in CD31 Tregs during rheumatoid arthritis progression. CD31 Y663F‐mutant Tregs exhibit a glucose‐to‐fructose metabolic shift, characterised by reduced glucose uptake and enhanced fructose utilisation regulated by PFKFB3. In contrast, Y686F mutation or CD31 KO disrupts glycolysis whilst preserving mitochondrial oxidative phosphorylation through RNF111/OGT pathway activation, sustaining Adenosine Triphosphate (ATP) production under metabolic stress.

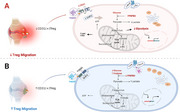

## 1 INTRODUCTION

CD31, also known as Platelet Endothelial Cell Adhesion Molecule‐1 (PECAM‐1), regulates both naive T cell access to secondary lymphoid tissue and inflammation‐induced T cell migration in vivo, exerting a complex influence on T cell trafficking that adapts to inflammatory conditions and molecular mechanism. T lymphocytes with high levels of CD31 expression exhibit increased aggregation at inflammatory sites, suggesting that CD31 enhances T cell migration by promoting intercellular adhesion and signal transduction.^1^, ^2^

Regulatory T cells (Tregs) play a pivotal role in preventing autoimmune disorders such as rheumatoid arthritis (RA) by suppressing aberrant immune activation and maintaining peripheral tolerance. In RA, impaired Treg migration and function contribute to synovial inflammation and joint destruction.^3^, ^4^ CD31 signalling is mediated mainly by two immunoreceptor tyrosine inhibitory motifs (ITIMs) in its cytoplasmic tail.^5^ These ITIMs are phosphorylated at Y663 and Y686 position upon CD31 engagement and subsequently serve as docking sites for protein‐tyrosine phosphatases including the Src homology 2 domain‐containing protein, SHP‐2.

Fructose metabolism is increasingly recognised as an adaptive mechanism that enables cells to rapidly produce and store energy, especially under conditions where glucose availability may be limited.^6^ Recent studies have suggested that CD31 also influences metabolic processes, particularly the utilization of fructose, an alternative energy source that may be crucial for Treg function during migration.^7^ In Tregs, the ability to switch to fructose metabolism can enhance their migratory capacity and functional efficacy.^8^ One of the key enzymes involved in this process is PFKFB3, which regulates the conversion of fructose‐6‐phosphate to fructose‐2,6‐bisphosphate, a potent activator of glycolysis. The activity of PFKFB3 is critical for Tregs to efficiently utilise fructose, thereby supporting their energy needs during migration.^9^

Additionally, the O‐GlcNAc transferase (OGT) and RNF111 proteins play significant roles in modulating Treg metabolism and migration.^10^, ^11^ OGT is involved in the post‐translational modification of proteins through O‐GlcNAcylation, which can influence various cellular processes, including signalling and metabolism.^12^, ^13^ RNF111, on the other hand, has been implicated in the regulation of the OGT activity and the downstream signalling pathways that affect the Treg function.^11^ The interplay between RNF111 and OGT is essential for maintaining mitochondrial function and facilitating the metabolic switch necessary for effective Treg migration.

This study aims to elucidate the relationship between CD31 and fructose metabolism in Tregs, particularly focusing on the roles of the ITIMs Y663 and Y686 in regulating this metabolic switch. By investigating how these residues influence the PFKFB3 activity, as well as the regulatory functions of OGT and RNF111, we seek to provide deeper insights into the metabolic requirements of Tregs during migration. Understanding these mechanisms is crucial, as they may offer novel therapeutic targets for autoimmune diseases, particularly RA, where Treg migration and function are often impaired.^14^ Our research could pave the way for innovative strategies to enhance Treg efficacy in autoimmune conditions by modulating their metabolic pathways.

## 2 RESULTS

### 2.1 CD31 promotes Treg cells migration and alleviates rheumatoid arthritis in patients and CIA mouse model

**FIGURE 1:**
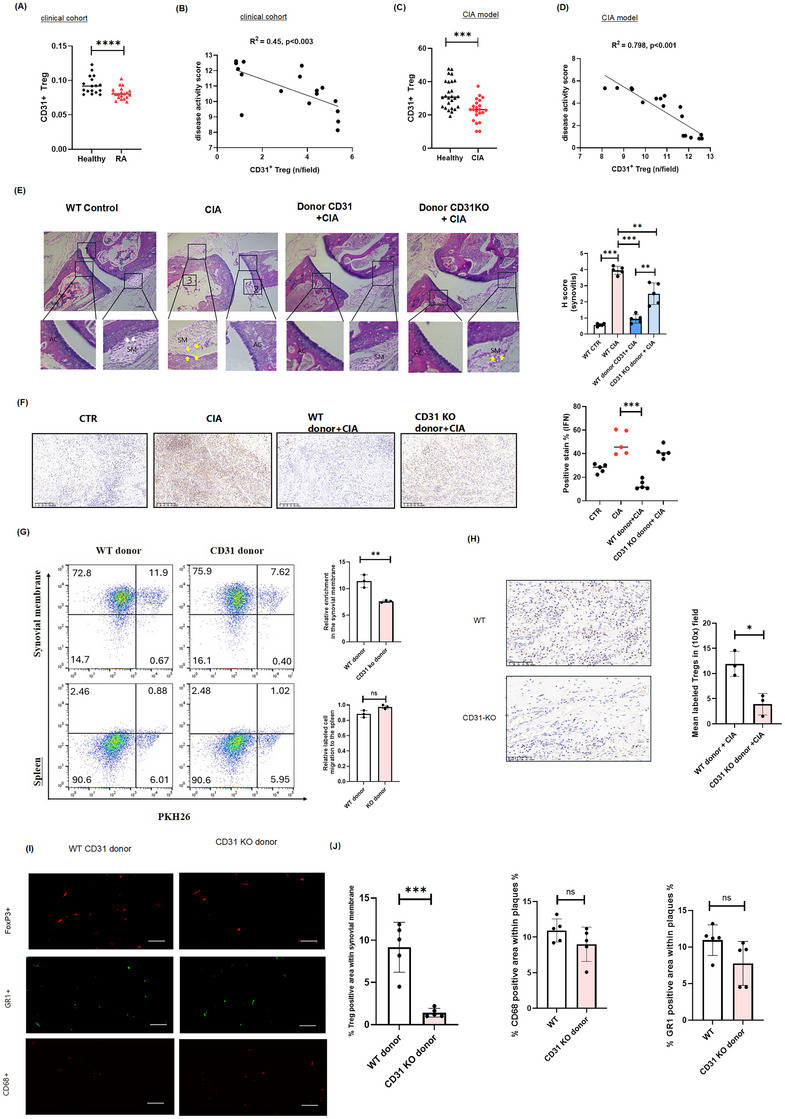
CD31 is required for Treg cell trafficking which alleviates RA in both patients and a mouse model. (A) Flow cytometry analysis the number of CD31+ Treg cells in healthy individuals and RA patients, histograms display the data results (*n* = 17 healthy subjects, *n* = 20 RA patients), The average ages for the two groups were 33.36 ± 7.54 and 38.62 ± 5.73 years old, respectively. *p < 0.05. (B) Scatterplot depicting the statistical correlation between the quantity of CD31+ Treg cells and disease activity score (*R* ^2 ^= 0.45). (C) The numbers of CD31+ Treg cells in healthy (*n* = 27) and CIA mice model (*n* = 22). (D) A negative correlation was observed between the number of CD31+ Treg cells and the disease activity score in the CIA mouse model (*R* ^2 ^= 0.798). **(E)** Haematoxylin and eosin (H&E) staining to assess the severity of synovitis, ex vivo Treg cells were isolated from CD31 KO mice and control mice, then injected intravenously into recipients with CIA possessing a RAG background. Box 1 reveals a reduced joint space compared to the arthritic control. Box 2 depicts notable lymphocytic infiltration in the synovium and surrounding bone invasion. Box 3 illustrates articular bone destruction. AC means articular cartilage; SM means synovial membrane. The bar chart on the right showed the H score. (F) Immunohistochemistry (IHC) was used to detect the expression level of Interferon (IFN)‐γin the synovium membrane of recipient CIA mice treated with CD31 WT Tregs or CD31 KO Tregs in vivo, scale bar represents 100 µm. Quantitative analyses were performed by counting 100 cells per sample. (G) Flow cytometry analysis reveals that CD31 KO donor Treg cells exhibit impaired migration to the synovial site, whilst migration to the spleen remains unaffected. (H) IHC images show the infiltration of WT vs. KO donor Treg cells into the synovial membrane. Scale bar, 50 µm. Analyses counted 500 cells per sample. (I,J) Immune cells, including CD68+ macrophages and GR1+ monocytes and granulocytes were isolated from CD31 WT or KO mice in vivo. These donor cells were then adoptively transferred to recipient mice with CIA, and the infiltration of these cells was assessed. A scale bar of 50 µm was used for reference, and analyses involved counting 500 cells per sample. Cumulative data from at least three samples (mean ± SD). Statistical significance was determined using a two‐tailed Student's *t*‐test, ns means nonsignificant, *p < 0.05, **p < 0.01 and ***p < 0.001. RA, rheumatoid arthritis; WT, wild‐type, KO, knockout.

To investigate the role of CD31+ Treg cells in RA, we isolated Treg cells from RA patients and healthy individuals in a clinical cohort study. Our results indicated that RA patients exhibited a reduction in the frequency of CD31+ Treg cells (Figure 1A). Regression analysis showed a strong negative correlation between the number of CD31+ Treg cells and RA disease severity (*R* ^2^ = 0.45, *p* < 0.003) (Figure 1B). We subsequently validated our findings in the Collagen‐Induced Arthritis (CIA) mouse model, revealing a relative lower amount of CD31 in the joint tissues of CIA mice compare to the healthy mice (*R* ^2^ = 0.798, *p* < 0.001), consistent with the results of the clinical cohort (Figure 1C,D).  To further explore this relationship, we intravenously injected CD31+ WT donor Treg cells and CD31 KO donor Treg cells into CIA recipient mice. Only CD31+ donor Treg cells, not CD31 KO donor Treg cells, were able to alleviate arthritis in recipient CIA mice (Figure 1E). We also observed that Interferon (IFN)‐gamma expression in the synovial membrane of recipient CIA mice exhibited a pro‐inflammatory response, whilst the infusion of WT CD31+ donor Treg cells led to a downregulation of IFN‐gamma expression, and was accompanied by significant statistical significance (Figure 1F). These results indicate that a reduction in CD31 cell numbers is associated with the progression of RA, and the infusion of CD31+ Treg cells may help reduce inflammation and alleviate RA symptoms.

We then assessed the infiltration of Treg cells in different tissues. Flow cytometry analysis revealed that CD31 KO donor Treg cells displayed impaired migration to the synovial site, whilst their migration to the spleen unaffected with no statistical significance (Figure 1G). Additionally, we validated our findings using immunohistochemistry (IHC) to compare wild‐type (WT) and CD31 KO donors in the synovial membrane of CIA mice, the results showed that the infiltration of Treg cells from CD31 KO donors into the synovial membrane was inhibited (Figure 1H).

To eliminate the possibility of off‐target effects resulting from changes in other immune cells, we isolated CD68+ for macrophages and GR1+ for monocytes and granulocytes from CD31 WT or KO mice. These cells were then adoptively transferred into syngeneic CIA recipients in a RAG1 KO background (RAG1 KO mice, which lack mature T cells and B cells). The results showed that the inhibition of infiltration by CD31 KO donor cells mainly occurred in Treg cells, but not in other immune cells (Figure 1I,J).

### 2.2 Regulating Treg migration through potential competitive inhibition between OGT and RNF111

CD31 activation initiates a signalling cascade characterised by the phosphorylation of specific intracellular tyrosine residues, which is essential for immune regulation and intercellular communication.^15^ A key event following CD31 activation is its interaction with SHP‐2, a critical phosphatase that undergoes autophosphorylation on its tyrosine site upon recruitment, which subsequently amplifying downstream signalling.^16^ This CD31–SHP‐2 axis plays a pivotal role in modulating signalling dynamics within Treg cells.

Our data show that stimulation with the chemokine CXCL11 enhances the physical interaction between CD31 and SHP‐2, as evidenced by immunofluorescence analysis (Supplementary Figure S1D). In line with these findings, Western blot and flow cytometry analysis demonstrated that both SHP‐2 and phospho‐tyrosine levels were significantly decreased in CD31 KO Tregs, confirming impaired downstream signalling in the absence of CD31 (Figure S1A–C). These observations are consistent with prior literature.^17^, ^18^

**FIGURE 2:**
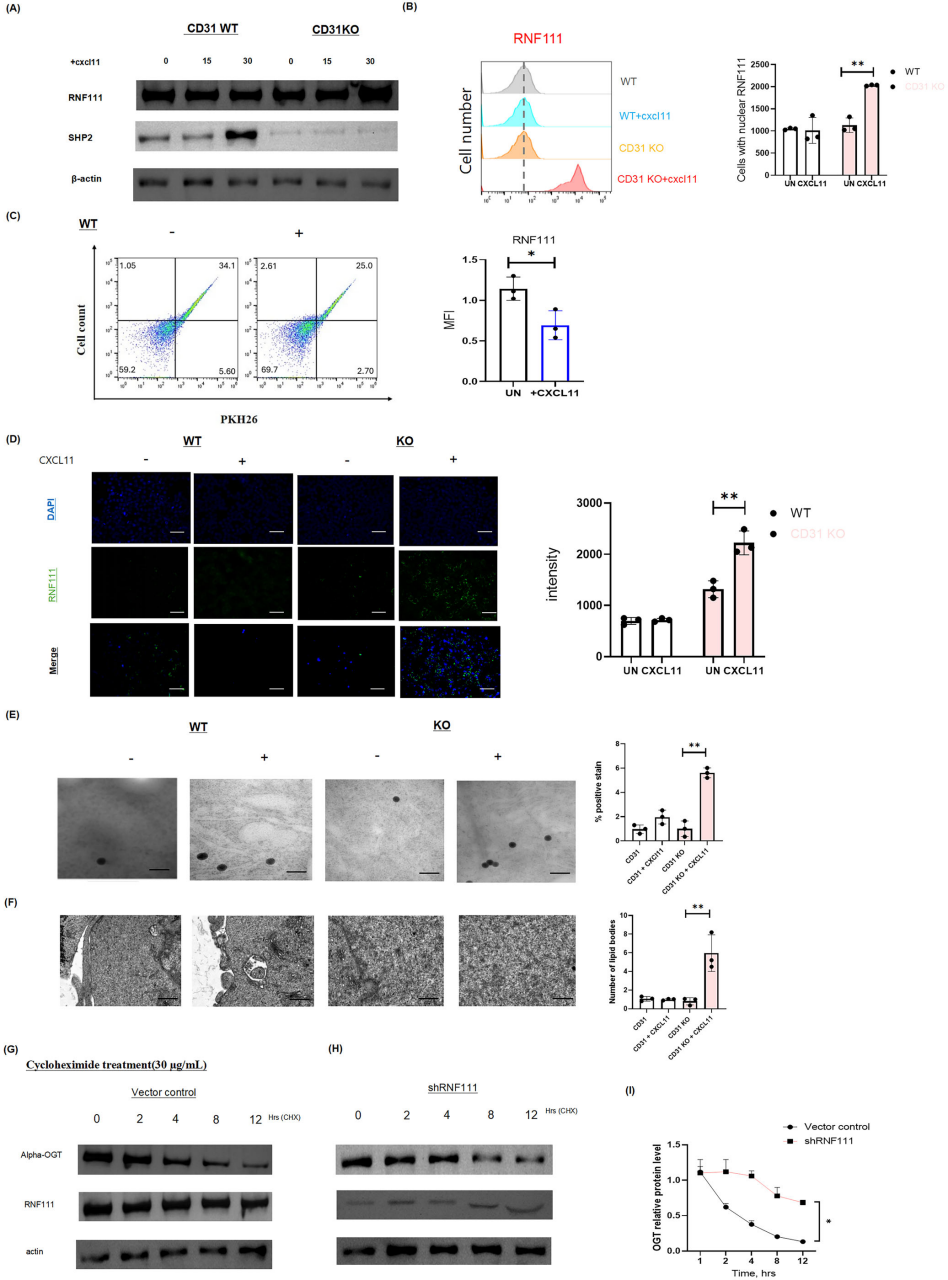
Regulating Treg migration by potential competitive inhibition between OGT and RNF111. (A) Expression of RNF111 and SHP‐2 in WT and CD31 KO ex vivo Treg cells exposed to proinflammatory CXCL11 at 0, 15 and 30 min. (B) Flow cytometry was used to detect the number of cells expressing RNF111 in WT Treg cells and CD31 KO Treg cells w/wo CXCL11 treatment. Bar chart shows the increase in CD31 KO treated with CXCL11. (C) Flow cytometry analysis of PHK26‐labelled Tregs isolated from the synovial membrane demonstrated inhibition of RNF111 expression exposed to CXCL11. The bar charts on the right illustrate fluorescence intensity of RNF111. (D) Immunofluorescence staining of RNF111 in WT or CD31 KO ex vivo Treg cells w/wo CXCL11. Scale bar, 50 µm. Analyses counted 500 cells per sample. (E) Transmission electron microscopy (TEM) imaging with immunogold labelling revealed the ultrastructural localisation of RNF111 in nanoparticles following in WT or CD31 KO ex vivo Treg cells w/wo CXCL11. The experiment was based on immunostaining using RNF111‐specific antibodies (scale bar: 500 nm). Bar chart shows the positive staining rate. (F) TEM shows the number of lipid droplets in CD31 or CD31 KO ex vivo Treg cells w/wo CXCL11 treatment for 4 h (scale bar: 500 nm), *n* = three independent experiments. (G,H) Western blotting analysis of OGT and RNF111 levels in vector control or RNF111 KO ex vivo Treg w/wo cycloheximide treatment (30 µg/mL CHX, 4 h), time points are indicated. (I) OGT relative protein level at different time points. Cumulative data from at least three samples (mean ± SD). Statistical significance: *p < 0.05, **p < 0.01, ***p < 0.001, **** *p* < 0.0001. OGT, O‐GlcNAc transferase; WT, wild‐type, KO, knockout.

Moreover, our results suggest that the CD31–SHP‐2 signalling axis exerts regulatory control over RNF111, an E3 ubiquitin ligase known to modulate protein function and signal transduction via ubiquitination.^19^ Specifically, CD31–SHP‐2 signalling appears to inhibit RNF111 activity in response to CXCL11 stimulation (Figure 2A). Taken together, these findings reveal a previously underappreciated link between CD31‐mediated tyrosine phosphorylation events and the ubiquitin‐proteasome pathway in Treg migration.

Flow cytometry analysis revealed that RNF111 expression is significantly elevated in CD31 KO cells compared to CD31 WT cells, indicating that the absence of CD31 leads to RNF111 upregulation (Figure 2B,C). Additionally, immunofluorescence analysis further confirmed that the deletion of CD31 enhances RNF111 expression under chemokine stimulation, highlighting the importance of metabolic regulatory interactions when CD31 is absent (Figure 2D).

To investigate the distribution and abundance of RNF111 within cells under WT and CD31 KO conditions, we examined cells stained with RNF111‐specific antibodies to visualise RNF111‐positive nanoparticles. Consistently, electron microscopy images show that RNF111 levels are increased in CD31 KO cells (Figure 2E). We subsequently investigated the changes in metabolic mechanisms in CD31 KO cells. Transmission electron microscopy (TEM) revealed that lipid droplet levels were significantly higher in CD31 KO cells compared to CD31 WT cells. The increased presence of lipid bodies in these cells indicates that Tregs are more predisposed to lipid metabolic pathways following CD31 deletion (Figure 2F). These observations further suggest that the upregulation of RNF111 specifically in CD31 KO ex vivo Tregs may represent a compensatory mechanism to adapt to altered metabolic conditions. Additionally, OGT modifications can influence the dynamics of actin and microtubules, thereby affecting cell morphology and migration.^20^ Our analysis using western blotting and qPCR quantification revealed a negative correlation between OGT and RNF111 expression, suggesting that RNF111 may act as an E3 ubiquitin ligase involved in the degradation of OGT (Figure 2G–I). This implies that CD31 deficiency elevates RNF111 levels, consequently reducing OGT protein stability. The depletion of OGT is likely to impair O‐GlcNAc‐mediated post‐translational modifications required for cytoskeletal reorganization, ultimately compromising Treg migratory capacity. The RNF111–OGT axis thus emerges as a critical regulatory mechanism for T cell motility under CD31‐deficient conditions, where enhanced RNF111 activity disrupts metabolic‐sensing pathways governing the cellular movement.

Furthermore, the PPI network analysis in the CD31 KO model reveals that OGT and RNF111 are interconnected with multiple nodes, including SIN3A, SUDS3, and several SAP family proteins. This suggests that the RNF111–OGT axis plays a significant role in influencing cellular metabolism and transcription by interacting with other amino acids following CD31 KO (Figure S2B). We next observed the changes in amino acid metabolomics between WT and CD31 KO cells.

In WT cells, CXCL11 treatment significantly increased the amino acid metabolic levels of aspartic acid, methionine, leucine and lysine (Figure S2A). This might indicate that CXCL11 promotes the metabolism or accumulation of these amino acids through some mechanism. Whilst the metabolic levels of amino acids such as alanine, lysine, and phenylalanine were markedly reduced in KO cells exposed to CXCL11, which might suggest that the metabolic levels of amino acids were altered by affecting the RNF111/OGT axis after CD31 knockout (KO).

### 2.3 shRNF111 in CD31 KO Tregs restored migration by upregulating the expression of signal transduction proteins

**FIGURE 3:**
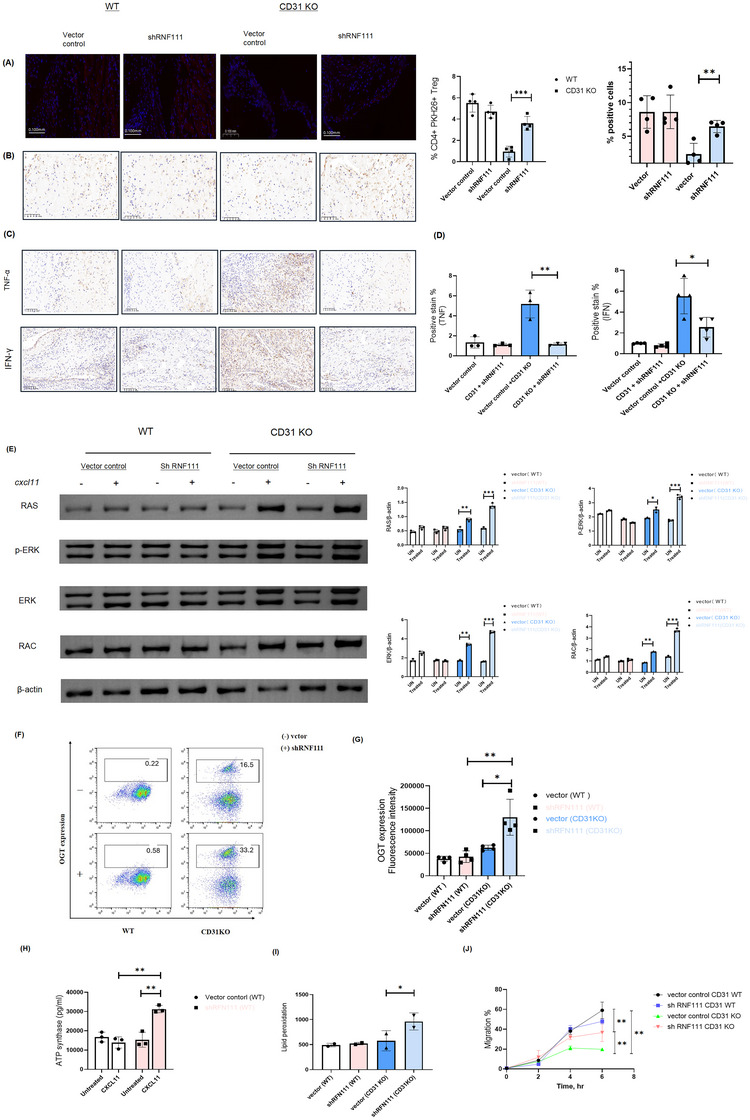
RNF111 and oxidative phosphorylation downregulate Treg migration in CD31 KO cells. (A) Confocal images show the CD4+ Treg infiltration of intravenously injected PKH26‐labelled WT or CD31 KO Treg cells (10⁷ cells per mouse) in recipient CIA mice with a RAG knockout background in shRNF111 or vector control group. (B) IHC reveals the expression of Foxp3 Treg migration in CD31 and CD31 KO Treg cells, with a scale bar of 50 µm. Analyses counted 500 cells per sample in shRNF111 or vector control group. (C,D) IHC was performed to evaluate TNF‐alpha and IFN‐gamma in vector/ shRNF111 intravenously injected CD31 WT or KO donor Treg. Representative images at 200× magnification were obtained for each tissue section from mice in each group, with a scale bar of 50 µm. Analyses counted 500 cells per sample. (E) Representative and summarised Western blot data for Ras, phosphorylated ERK (p‐ERK), total ERK and RAC in CD31 WT or KO donor Treg cells, with or without CXCL11, following vector or shRNF111 treatment. (F,G) Flow cytometry illustrates the expression of OGT in CD31WT and CD31 KO Treg following vector or shRNF111 treatment. (H) The metabolic activity of ATP synthase in vector control/shRNF111 Tregs w/wo CXCLl11.The bars in the graphs represent the mean values plus standard deviation. Statistical significance is denoted as follows: *p < 0.05, ** *p* < 0.01, ***p < 0.001. (I) The levels of lipid peroxidation after vector control/shRNF111 Tregs treatment in CD31 and CD31 KO group. (J) An in vitro transendothelial migration assay was conducted comparing CD31 WT and KO cells, both with and without shRNF111 Treg. Mice were received an intravenous injection of PKH26‐labelled WT or CD31 KO Treg cells (10^7^ cells per mouse) after 2 days. WT, wild‐type, KO, knockout; IHC, immunohistochemistry.

To further investigate the role of RNF111 in Treg cell migration, we conducted an in vitro migration assay using PKH26‐labelled WT or CD31KO Treg cells, which were intravenously injected into recipient CIA mice with a RAG KO background. Confocal images revealed that CD31 KO Treg cells exhibited reduced migration compared to WT cells. Notably, RNF111 knockdown by shRNA in CD31 KO Tregs largely restored migration (Figure 3A,B). Immunostaining further revealed decreased expression of TNF‐alpha and IFN‐gamma in RNF111 KO Treg cells, partially mitigated the inflammatory response induced by CD31 KO (Figure 3C,D).

Subsequently, we focused on several key markers involved in regulating Treg cell migration and function during immune responses, including Ras, phosphorylated ERK (p‐ERK), total ERK and RAC, to assess their alterations in the context of RNF111 KO. The increase in these proteins upon RNF111 KO indicates that RNF111 influences Treg cell migration by regulating the expression of these signal transduction proteins (Figure 3E). Furthermore, flow cytometry analyses demonstrated that RNF111 KO in CD31 KO cells significantly increased OGT expression (Figure 3F,G).

Finally, we assessed the impact of shRNF111 on cellular metabolism and found that ATP synthase levels increased in RNF111 KO cells in response to CXCL11. This suggests that ATP metabolism in RNF111 KO cells may rely on oxidative phosphorylation (Figure 3H). Additionally, the increase in lipid peroxidation reflects the cellular state under oxidative stress and may indicate a shift in metabolic pathways (Figure 3I). To explore the role of RNF111 on mediated oxidative phosphorylation on migration, RNF111 was knockdown by shRNA in CD31 KO Tregs, which rescued migration (Figure 3J). These findings suggest a complex interplay between CD31, RNF111, and metabolic regulation in controlling Treg cell motility.

### 2.4 Non‐redundant effects of Y663F and Y686F mutants on the migration of Treg cells both in vitro and in vivo

**FIGURE 4:**
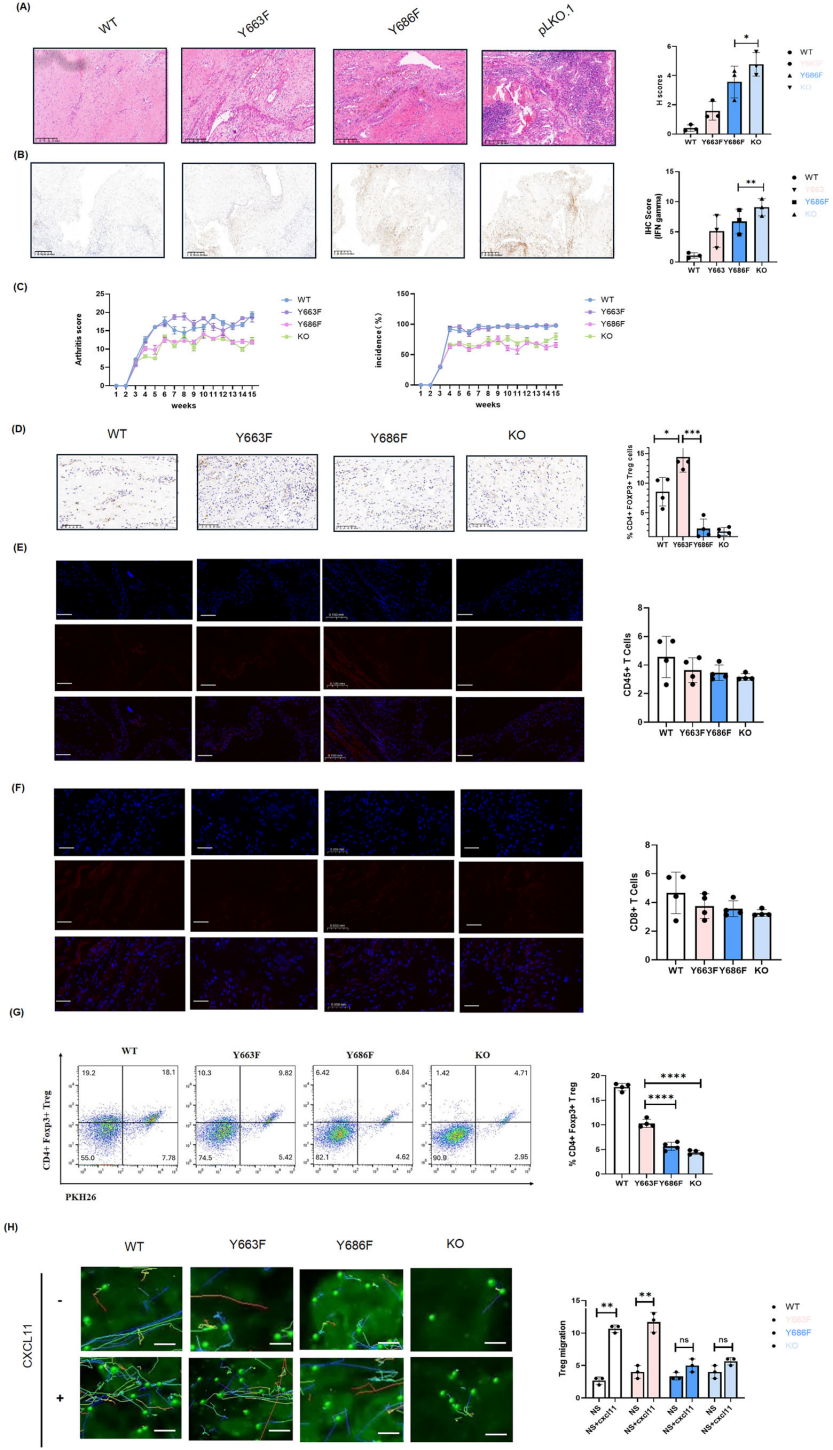
Nonredundant role of Y663F and Y686F mutants on Treg cell migration in vitro and in vivo. We performed site‐directed mutagenesis to replace the tyrosine residue at position Y663 and Y686 with phenylalanine and transduced w/wo CXCL11 treatment, subsequently ex vivo iv‐injection into recipient CIA in a RAG1 KO background. (A,B) After inducing CD31 Y686F and Y663F mutant Tregs, only the Y686F mutant donor Treg cells exacerbated arthritis similarly to CD31 KO Treg cells, as observed in both H&E staining and immunohistochemistry of INF‐gamma. pLKO.1 indicates cells transduced with the empty vector backbone. Scale bar, 100 µm. Analyses included 500 cells per sample in recipient CIA mice. (C) Arthritis scores and the ratio of disease incidences were recorded at indicated time points in each group. (D) Immunohistochemistry (IHC) showed the percentage of CD4+PHK26+ Treg cells in vector, Y663F, Y686F and pLKO.1 transduced. Analyses included 500 cells per sample measured in three independent experiments ± SD. Scale bar = 50 µm. (E,F) Confocal microscopy observed the numbers of CD45+ T cells and CD8+ T cells with CXCL11 in the synovial membrane in each group. Scale bar, 50 µm. Analyses included 500 cells per sample. (G) Fluorescence‐Activated Cell Sorting (FACS) analysis was conducted to measure PHK26 labelled CD4+Foxp3+ Treg cells infiltrating the synovial membrane in WT, Y663F, Y686F, and KO group exposed to CXCL11. (H) Treg migration was observed in WT‐, Y663F‐, Y686F‐ and KO‐group after untreated or exposed to the pro‐inflammatory chemokine CXCL11 (10 µM) for 6 h. Cumulative data from at least three samples (mean ± SD). Statistical significance: *p < 0.05, **p < 0.01, ***p < 0.001, **** *p* < 0.0001. H&E, Haematoxylin and Eosin; WT, wild‐type, KO, knockout.

To investigate the role of ITIM domains in CD31 on Treg migration, we generated several gene constructs: CD31 WT, CD31 pLKO.1 (CD31 KO), CD31Y663F, and CD31Y686F. Through site‐directed mutagenesis, we replaced the tyrosine (Y) residues at positions Y663 and Y686 with phenylalanine (F).^16^ We then adoptively transferred WT, Y663F mutant, Y686F mutant, and CD31 KO Treg cells into recipient mice with CIA. Histological analysis, scored using established criteria,^21^ revealed that WT and Y663F Treg cells ameliorated disease severity, as evidenced by improved histological scores (Figure 4A). Conversely, mice receiving Y686F and CD31 KO Treg cells exhibited exacerbated arthritis. This correlated with elevated synovial IFN‐γ levels, potentially driven by TNF‐α and other pro‐inflammatory cytokines^22^, ^23^ (Figure 4B,C).

Immunostaining of synovial tissue revealed that the infiltration of Y686F and CD31 KO donor regulatory T cells (Tregs, CD4+FOXP3+) was impaired. However, the infiltration of CD8+ T cells and total donor leukocyte (CD45+) remained unaffected (Figure 4D–G). Furthermore, confocal microscopy confirmed enhanced synovial infiltration of WT and Y663F Treg cells in vivo (Figure 4H), supporting the importance of these residues and CD31 in Treg cell trafficking and suppressive function in arthritis.

We previously demonstrated that glucokinase (GCK) promotes cytoskeletal rearrangements by associating with actin during Treg migration.^24^ Protein and gene expression analyses corroborated these findings, revealing reduced migratory capacity in Y686F and CD31 KO Treg cells compared to WT and Y663F cells (Figure S3A,B). Importantly, oxygen consumption rate (OCR) with a Seahorse analyzer, revealed significant enhanced in mitochondrial function in the Y686F and KO models (Figure S3C).

We then explored how mitochondrial function switch to in the absence of CD31 signals.^7^, ^16^ Electron microscopy revealed a profound impact of CD31 on Treg cell mitochondrial dynamics. Y686F and CD31 KO Treg cells exhibited an increased number of mitochondria, suggesting potential compensatory mechanisms for enhanced mitochondrial function (Figure S3D,E). Consistently, using 3D electron tomography and segmentation, we observed increased mitochondrial morphology and number in CD31‐KO Tregs. These changes included not only alterations in mitochondrial ultrastructure but also dynamic processes such as fission and fusion, indicating a fundamental shift in mitochondrial homeostasis (Figure S3F). Our data suggest that CD31 signalling is intricately linked to the maintenance of mitochondrial integrity and migration function in Treg cells.

### 2.5 Y663F mutation causes a shift from glucose to fructose metabolism by downregulating PFKFB3

**FIGURE 5:**
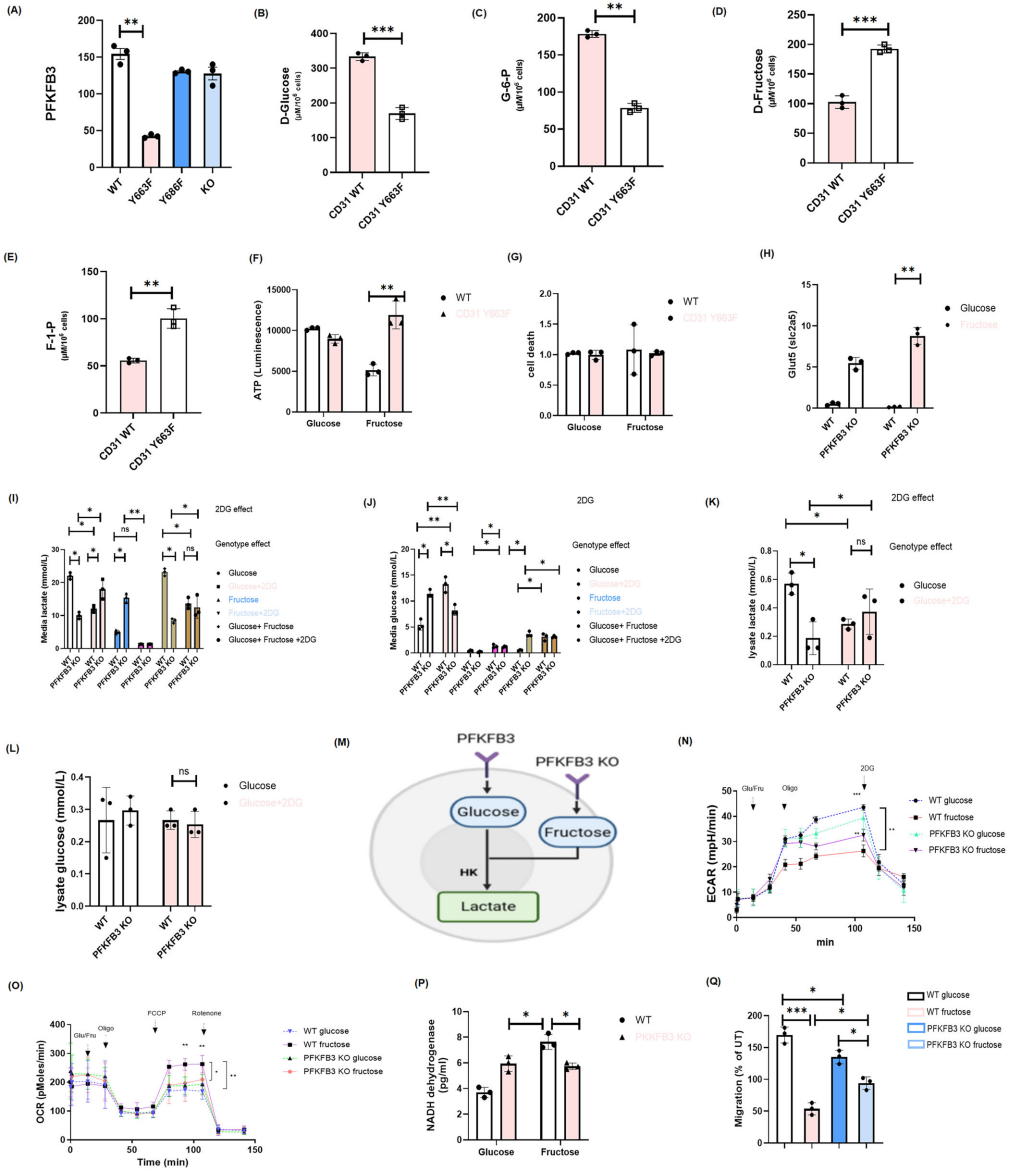
Comprehensive analysis of PFKFB3 regulation and metabolic pathways in CD31 WT and Y663F ex vivo Treg cells. (A) PFKFB3 mRNA expression was measured by qPCR in CD31 WT, Y663F mutant, Y686F mutant and KO cells. (B,C) ^13^C6‐glucose isotope tracing analysis was conducted to quantify glycolysis‐related metabolites, including glucose uptake (B) and glucose‐6‐phosphate (G6P) production (C), comparing CD31 WT and Y663F mutant Treg cells. (D,E) Cells were cultured in glucose‐free DMEM medium supplemented with 13C6‐fructose and 10% dialysed FBS for 12 h. Fructose uptake (D) and fructose‐1‐phosphate (F1P) levels (E) were analysed, highlighting metabolic differences between CD31 WT and Y663F mutant cells. (F,G) ATP levels in WT and Y663F mutant cells were measured after incubation with 25 mM glucose or 1 mM fructose for 12 h (F), and cell viability was assessed using the trypan blue exclusion assay (G). (H) GLUT5 mRNA expression was measured by qPCR in WT and PFKFB3 KO cells. (I,J) The bar graph shows the levels of lactate (I) and glucose (J) in the media of WT and PFKFB3 KO ex vivo Treg cells under various conditions: 2 mM glucose, glucose + 2DG, 10 mM fructose, fructose + 2DG, glucose + fructose, and glucose + fructose + 2DG. (K,L) The bar graph shows the (K) lactate production rate (in mmol/L) and (L) glucose consumption rate (in mmol/L) in the lysates of WT and KO ex vivo Treg cells under two conditions: glucose and glucose + 2DG. (M) The schematic illustrates that CD31 donor WT cells inhibit fructose‐mediated glycolysis and enhance glucose‐mediated glycolysis through PFKFB3. In PFKFB3 KO Tregs, fructose metabolism is primarily dominant, allowing more fructose to enter the glycolytic pathway and be converted to lactate by hexokinase (HK). (N,O) Seahorse extracellular flux analyzer measured real‐time glucose and fructose utilization by WT and PFKFB3 KO ex vivo Treg cells. Glucose (25 mM) or fructose (25 mM), oligomycin (1 µM) and FCCP (1 µM) were added at the indicated time points as illustrated in the figure. (P) Bar chart shows increased Nicotinamide Adenine Dinucleotide (NADH) dehydrogenase expression in PFKFB3 KO ex vivo Treg cells. (Q) Measurement of Treg migration by WT and PFKFB3 KO cells under glucose and fructose conditions. *N* = Three independent experiments, data are represented as mean ± SD. Statistical significance was denoted as follows: *p < 0.05, **p < 0.01, ***p < 0.001, ****p < 0.0001. “ns” denotes non‐significant difference. WT, wild‐type, KO, knockout.

6‐Phosphofructo‐2‐kinase/fructose‐2,6‐bisphosphatase 3 (PFKFB3) is a key enzyme involved in regulating glycolysis and glucose metabolism. By modulating the levels of fructose‐2,6‐bisphosphate, PFKFB3 influences the rate of glycolysis and energy production. Inhibition of PFKFB3 shifts the energy source of endothelial cells from glucose to fructose.^25^ Our prior research has demonstrated that the migration of Treg cell is dependent on glycolysis.^24^ Thus, we aimed to further explore why the migration of Treg cells and glycolytic pathway remains unaffected under the CD31 Y663F mutation, we continued utilising the CD31 Y663F Treg cellular model. Given that PFKFB3 serves as a critical mediator of energy metabolism, bridging glycolytic flux and energy availability, we analysed its expression levels across different groups. As anticipated, PFKFB3 expression was reduced in CD31 Y663F cells (Figure 5A). Consequently, utilising ^13^C6‐glucose as a metabolic tracer in a glucose‐free Dulbecco's Modified Eagle Medium (DMEM) medium, we examined the differences in energy source utilisation between CD31 Y663F Treg cells and control cells (Figure 5B,C). We observed a decreased uptake of glucose and a reduction in the production of G6P in CD31 Y663F Treg cells. In contrast, metabolic tracing experiments with ^13^C6‐fructose revealed a notably enhanced uptake of fructose and increased fructose‐1‐phosphate (F1P) production in CD31 Y663F Treg cells compared to controls (Figure 5D,E). These findings collectively suggest a compensatory metabolic shift towards fructose utilisation under conditions of reduced PFKFB3 expression induced by the CD31 Y663F mutation.

Treatment of Treg cells with glucose or fructose revealed that ATP production remained stable in CD31 Y663F Treg, and their survival was unaffected, indicating metabolic adaptability through enhanced fructose utilisation (Figure 5F,G). Moreover, GLUT5 expression increased after PFKFB3 KO under fructose conditions, reflecting enhanced fructose uptake as a metabolic compensation mechanism (Figure 5H).

Next, we measured lactate and glucose levels in the culture medium to evaluate metabolic alterations in WT and PFKFB3 KO Treg cells under conditions of glucose/fructose with or without 2DG. PFKFB3 KO cells exhibited lower lactate production and increased glucose accumulation under glucose conditions, confirming disrupted glycolytic metabolism. In contrast, 2DG treatment markedly reduced lactate production and led to glucose accumulation, confirming inhibition of glycolysis. Under fructose conditions, PFKFB3 KO cells produced significantly more lactate compared to WT cells, but 2DG treatment strongly suppressed lactate production in both cell types, suggesting reliance on fructose as an alternative glycolytic substrate. Mixing glucose and fructose substrates yielded intermediate lactate and glucose levels, suggesting combined metabolic effects (Figure 5I,J). Additionally, we analysed cell pellet lactate and glucose concentrations in glucose‐treated conditions, which matched those of the supernatant, further highlighting the role of PFKFB3 in glucose metabolism and cellular responses to metabolic stress (Figure 5K,L). We propose that fructose compensatory metabolism in PFKFB3 KO cells leads to increased lactate generation via fructose phosphorylation, as illustrated in the diagram (Figure 5M).

To further delineate the metabolic profiles, extracellular acidification rate (ECAR) and OCR were assessed. ECAR increased in response to glucose in both WT and KO cells. However, only PFKFB3 KO cells exhibited enhanced ECAR upon fructose exposure, confirming increased fructose utilisation (Figure 5N). Furthermore, OCR and NADH production significantly decreased in PFKFB3 KO cells under both substrates, suggesting reduced oxidative phosphorylation capacity due to limited glycolytic flux (Figure 5O,P). Assessment of Treg migration showed partial restoration in fructose‐treated PFKFB3 KO cells, indicating fructose metabolism partially compensates the impaired glycolytic function and supports migration (Figure 5Q). These results indicate that Y663F regulates metabolic flexibility by inhibiting PFKFB3, highlighting the intricate metabolic adaptations in Y663F.

### 2.6 Y686F mutation in CD31 by downregulating glycolysis to impair Treg migration

**FIGURE 6:**
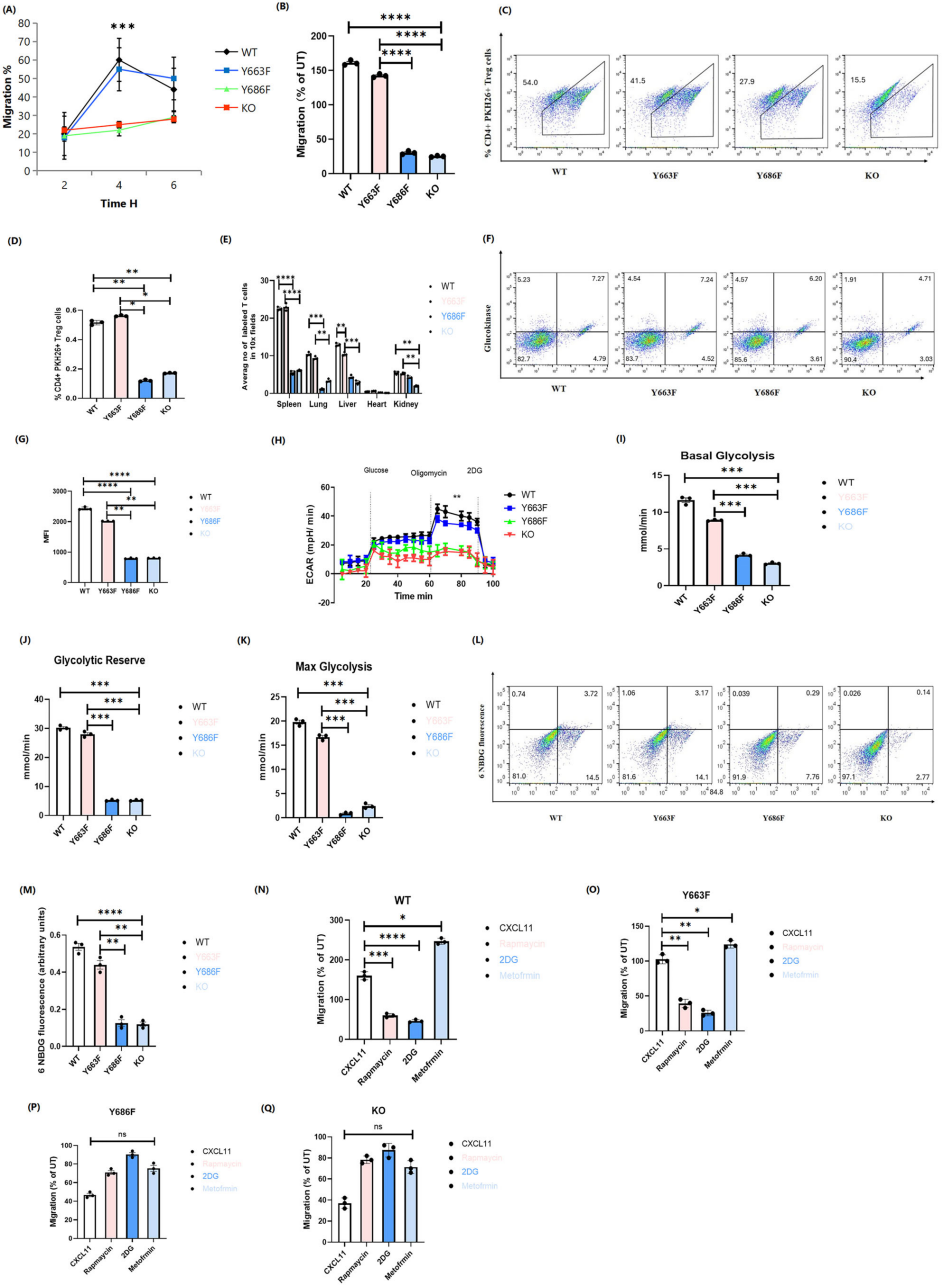
Y686F inhibits activated Treg cells transmigration both in vitro and in vivo, and Y686 Treg migration is dependent on basal and chemokine‐induced aerobic glycolysis. (A,B) Trans‐well chemotaxis assays of in vitro activated CD31 WT, KO, and Y663F and Y686F transgenic Treg cells towards CXCL11 (300 ng/mL) were shown as kinetics at the 4‐h time point. (C,D) Flow cytometry revealed Y686F and CD31 KO impaired migration of Treg cells to secondary lymphoid tissue in vivo. (E) The average number of labelled T cells in various tissues across groups, with data expressed as mean ± standard deviation (SD). Statistical significance is indicated as follows: *p < 0.05, **p < 0.01, ***p < 0.001, ****p < 0.0001. (F,G) Flow cytometry measured expression of Glucokinase in activated donor Treg cells treated with CXCL11 (1000 ng/mL). Histogram shown mean fluorescence intensity (MFI) of FACS, *N* = 3. (H) ECAR showed an inhibited glycolytic activity between Y686F and CD31 KO, whilst Y663F is unaffected. (I–K) Measurements of basal glycolysis, glycolytic reserve, and maximum glycolysis. (L,M) Glucose uptake and glycolytic flux in activated cells pretreated with the fluorescent probes 6‐NBDG. (N–Q) In vitro chemotaxis of activated cells pretreated with Rapamycin (200 nM), 2‐DG (1 mM), or Metformin (2 mM) and CXCL11 (300 ng/mL) over 4 h. Cumulative data from at least three animals, values denote (mean ± SD). *p < 0.05, ** *p *< 0.01, ***p < 0.001, ****p < 0.0001. WT, wild‐type, KO, knockout; ECAR, extracellular acidification rate.

We next sought to establish whether Y663F or Y686F activity is sufficient to maintain Treg migration during activation in physiological immune responses. We performed in vitro chemotaxis assays. The Y686F mutant, unlike the Y663F mutant, exhibited significant defects in the migration of activated Tregs, similar to CD31 KO cells (Figure 6A,B). Y686F or CD31 KO donor Treg showed significantly impaired migration to lymph nodes (Figure 6C,D) and impaired ability to access non‐lymphoid, non‐antigenic, and non‐inflamed tissues in vivo (Figure 6E). Mechanically, WT and Y663F donor Treg GCK expression was increase whilst Y686F mutant and CD31 KO significantly impaired, suggesting that the Y686F mutation may disrupt metabolic response mechanisms (Figure 6F,G).

To confirm the nonredundant migration role of Y663F in Treg migration, we measured the ECAR using a Seahorse analyzer. The results indicated that the Y686F mutation severely impaired glycolysis in CD31‐associated Treg cells (Figure 6H). Measures of basal glycolysis, glycolytic reserve, and maximal glycolysis were significantly downregulated in Y686F mutant and CD31 KO (Figure 6I–K). These findings were validated by a glucose uptake assay using fluorescently labelled 6‐NBDG (Figure 6L,M). Similar to our previous observations in Treg migration rely PI3K‐mTORC2‐ mediated pathway culminating in induction of GCK,^24^ we treated Treg cells with rapamycin (an mTOR inhibitor), 2‐DG (a glycolysis inhibitor), or metformin (a glucose uptake activator) and conducted in vitro chemotaxis assays towards CXCL11 for 4 h (Figure 6N–Q). The glycolytic dependency of Y686F mutation is impaired.

### 2.7 Characterization of CD31‐mediated regulation of RNF111 and metabolic changes in Treg cells isolated RA

**FIGURE 7:**
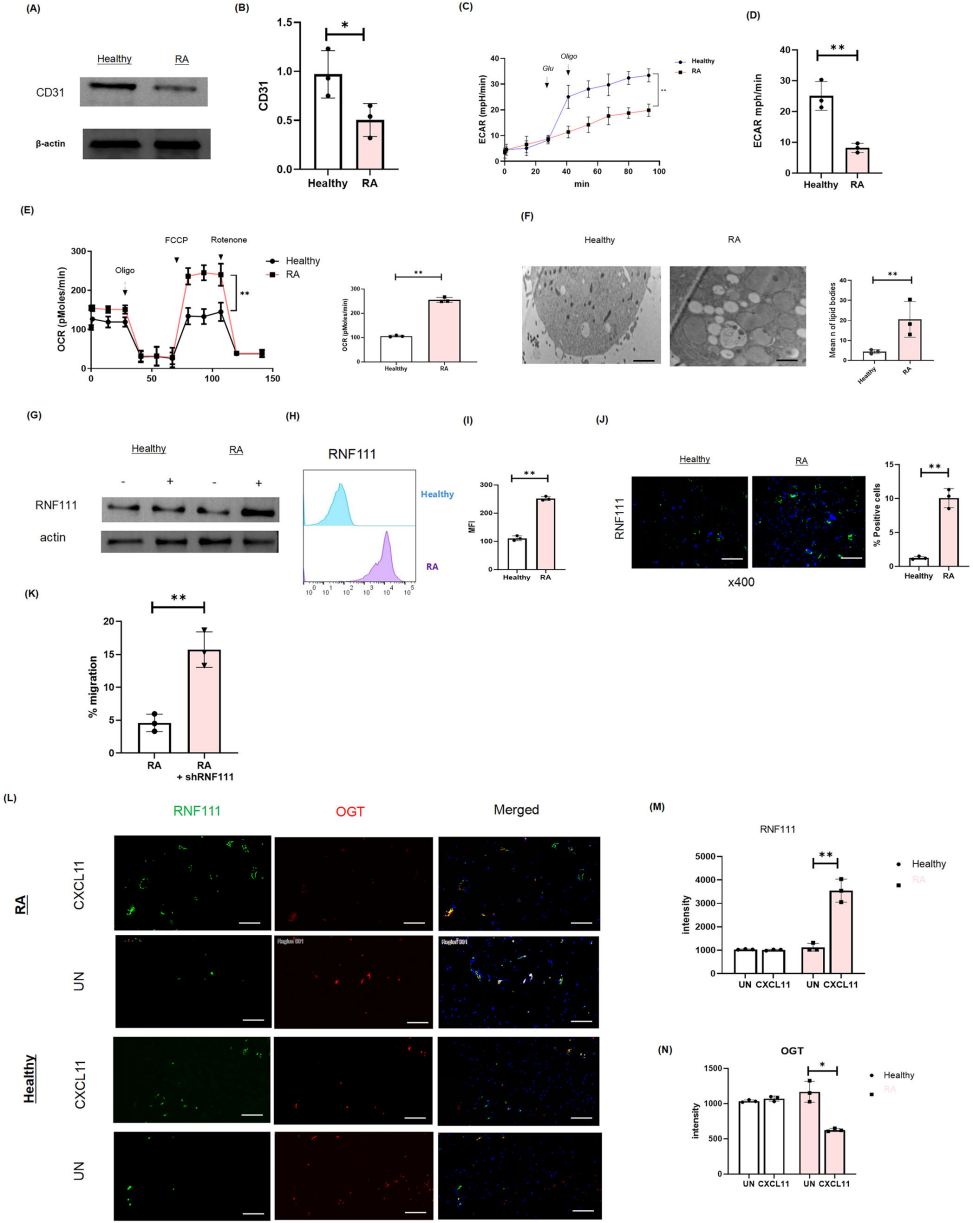
Increase expression of RNF111 and lipid bodies in Treg cells isolated RA. (A,B) Western blot analysis of CD31 expression in Treg cells isolated from healthy individual vs. RA synovial fluid. (C,D) ECAR measurements in Treg cells isolated from healthy individual vs. RA synovial fluid. (E) OCR is increased in Treg cells from RA patients compared to Treg cells isolated from healthy controls. (F) TEM images show increased lipid bodies in Treg cells isolated from RA patients. Scale bar represents 500 µm. A total of 500 cells were counted. (G) Increased expression of RNF111 in Treg cells isolated from RA as demonstrated by Western blot. (H,I) FACS measurement of RNF111 in Treg cells isolated from RA and healthy individuals, **p < 0.01. (J) Immunostaining shows increased RNF111 in Treg cells isolated from RA. Scale bar represents 25 µm. Analyses included 500 cells per sample. (K) Migration measurement in Treg cells isolated from RA with or without shRNA‐mediated knockdown of RNF111. (L–N) Immunofluorescence revealed the localisation of RNF111 and OGT in Treg cells isolate from healthy individuals and RA patients, with and without CXCL11. The bar chart showed the fluorescence intensity of OGT and RNF111. Analyses counted 500 cells per sample with a scale bar of 50 µm. Data are cumulative from at least three independent experiments and are presented as mean ± SD. Statistical significance is indicated as follows: * *p* < 0.05, **p < 0.01, ***p < 0.001, ****p < 0.0001.RA, rheumatoid arthritis; OCR, oxygen consumption rate; TEM, transmission electron microscopy.

Given that healthy Treg cells display higher CD31 expression compared to Tregs isolated from patients with RA (Figure 7A,B), we further characterised the role of glucose metabolism and RNF111 in Treg cell migration by exposing the cells to CXCL11. As expected, Tregs from individuals with RA showed attenuated glycolysis (Figure 7C,D). In addition, our study of metabolic pathways found that RA Tregs displayed enhanced mitochondrial function alongside compensatory lipid body formation, suggesting a metabolic shift towards lipid storage and oxidative phosphorylation as an adaptive response to impaired glycolysis (Figure 7E,F). Furthermore, we also found RNF111 was highly expressed in Treg cells in RA patients, as demonstrated by Western blot analysis and cellular localisation studies (Figure 7G–J). This may be related to the impaired migration of Tregs in RA patients.^26^ It also further confirms our previous findings that the expression of RNF111 increased after CD31 KO. Notably, knocking down RNF111 with siRNA partially restored impaired migration in RA Tregs, implicating RNF111 in cytoskeletal and chemotactic regulation (Figure 7K). Consequently, we used confocal imaging to measure the co‐localisation of RNF111 and OGT in Tregs from healthy individuals and RA patients. Intensity quantification revealed a decrease in OGT expression and an increase in RNF111 levels in RA Tregs, particularly under stimulation with CXCL11, suggesting a chemokine‐dependent crosstalk. The low OGT activity in RA may fail to counterbalance the RNF111‐mediated ubiquitination, resulting in Treg migration failure and persistent inflammation in RA (Figure 7L–N).

## 3 DISCUSSION

Our research aligns with previous studies in both murine and human systems, which have demonstrated that CD31 sustains the development of specific T cell infiltrates and pathology in various experimental models of disease. The migration of Tregs plays a crucial role in this process. Reinstating physiological self‐tolerance by boosting the inherent suppression of pathogenic autoreactive T cells by Tregs is an effective strategy for treating RA.^27^ In the CIA model, the injection of transferred WT and Y663F donor Tregs reduced disease severity and slowed disease progression.

Our study elucidates the distinct roles of CD31 Y663 and Y686 residues in regulating Treg cell migration through distinct metabolic pathways. We discovered that PFKFB3 inhibition by WT CD31 cells, thereby limiting fructose‐mediated glycolysis. However, the Y663F mutation significantly reduces this suppression, allowing for enhanced fructose metabolism. This metabolic shift is characterised by increased fructose‐mediated glycolysis, which restores energy levels necessary for Treg migration. The upregulation of PFKFB3 in CD31 Y663F cells further supports this metabolic adaptation, facilitating increased transcellular fructose transport.

Furthermore, seahorse assays revealed that whilst fructose is not the primary fuel in Y663F cells, it still effectively downregulates oxidative phosphorylation to a degree comparable to PFKFB3 inhibition in WT Treg. This indicates that the Y663F mutation enables Tregs to utilise fructose efficiently, thereby maintaining energy production and supporting cell migration. In contrast, the Y686F mutation presents a different metabolic profile. The decrease in fructose/glycolysis pathways suggests that Y686F donor T regs preferentially switch to oxidative phosphorylation. This shift is associated with impaired Treg migration, as evidenced by the lack of glycolytic reserve in KO Treg when exposed to fructose medium. The elevated OXPHOS in Y686F and KO cells highlights their inability to downregulate PFKFB3 expression, resulting in decreased fructose‐mediated glycolysis. Interestingly, studying the transcriptional regulation of GLUT5 and PFKFB3 and exploring the potential involvement of noncoding RNAs in linking these mediators of metabolism may offer additional insights and therapeutic targets for yet‐to‐be‐uncovered clinical applications.^28^ Mechanically, our results show significant switch to the mitochondrial function in the Y686F and in the absence of CD31. These findings reinforce the importance of glycolysis in Treg cell migration and highlight the crucial role of CD31 signalling in regulating both cell motility and energy metabolism.

The acquired Treg migration to Y663F CD31:SHP‐2 is the activation of the Rac1 and Rock1 pathway. Hence, Y663F is a key regulator in the Rho GTPase pathway affecting Treg migration. This also highlights the regulatory interplay between RNF111 and OGT enzymes in modulating Treg cell migration. Other studies have primarily implicated RNF111 in the transforming growth factor‐beta (TGF‐β) and bone morphogenetic protein (BMP) signalling pathways.^29^ This is a previously unknown pathway for the acquisition of immune migration the impact of RNF111 on OGT expression. Subsequently, OGT modification further influences the transcriptional regulation of migration‐related proteins on Treg cells with alterations in specific amino acids being crucial for this process. We also conducted Gene Ontology (GO) analysis revealed significant enrichment of pathways related to amino acid metabolism, Treg cell regulation, and increased lipid metabolism, with upregulated RNF111 implicated in these changes.

RNF111 and OGT critically regulate Treg migration and energy adaptation through distinct mechanisms. RNF111, an E3 ubiquitin ligase, maintains metabolic enzyme stability via ubiquitination. When the CD31 Y686F mutation disrupts glycolysis–fructolysis switching, RNF111 compensates by enhancing mitochondrial oxidative phosphorylation to sustain energy production. OGT, through O‐GlcNAcylation, dynamically modulates signalling networks and metabolic flux, enabling rapid adaptation to energy demands during migration. The Y686F mutation specifically activates this RNF111/OGT axis, revealing their compensatory role when glycolytic–fructolytic switching fails. Specifically, whilst Y663 supports fructose metabolism, Y686 recruits RNF111/OGT to preserve the mitochondrial function. This pathway choice underscores their dual function in maintaining energy homeostasis and adapting to CD31 signalling defects making them mechanistic linchpins in Treg migration and therapeutic targets for RA.

Moreover, our study emphasised that patients clinically diagnosed with RA, the infiltration of CD31+ Tregs decreased in the synovium. Thus, it might be worthwhile alleviated the disease by increasing the number of CD31+ Treg cells, suggesting a novel perspective for investigating cell migration mechanisms and potential therapeutic approaches for RA. In addition, CD31 has been implicated in the development of atherosclerosis and its clinical complications.^30^, ^31^ Several studies have identified a connection between specific CD31 single nucleotide polymorphisms (SNPs) and the development of atherosclerosis, suggesting that variations in CD31 may influence immune responses and vascular health.^32^ In summary, our findings highlight the critical role of CD31+ in modulating Treg metabolism and migration, particularly through the regulation of glucose metabolism and glycolysis. This metabolic adaptation is essential for maintaining immune tolerance in the process of RA, underscoring CD31+ as a significant therapeutic target for enhancing immune regulation and treatment outcomes.

## 4 MATERIALS AND METHOD

### 4.1 Animal experiment

All the animal experiments were reviewed and approved by the Ethics Committee of Hong Kong Baptist University (REC/20–21/0584). All animal experiments conducted in this study were complied with the WMA Statement on animal use in biomedical research. We injected the hybrids of 150 µg of chicken CII (Sigma) with equal volume of Freund's complete adjuvant containing 200 µg of H37Ra Mycobacterium tuberculosis (BD Biosciences, cat. no. 231141) intradermally at the base of the tail of the mice to artificially induce collagen‐induced arthritis (CIA).^16^

### 4.2 Clinical materials

The human studies were all approved by the Ethics Committee of Shanghai Sixth People's Hospital in accordance with the World Medical Association's Declaration of Helsinki and gave written informed consent for this study. RA patients met the 2010 American College of Rheumatology (ACR) classification criteria.^33^

### 4.3 Treg preparation and in vitro chemotaxis assay

Tregs were isolated from lymph nodes and spleen in WT (C57BL/6), Y663F, Y686F and CD31 KO mice. Resuspend cells in the phosphate‐buffered saline (PBS) medium at a concentration of 1 × 10^6^ cells/mL. Use a Transwell chamber with a pore size of 5 µm and place the chamber in a 24‐well plate. Treg cell were activated by anti‐CD3 and anti‐CD28 antibodies; prepare a gradient by adding the pro‐inflammatory chemokine CXCL11(300 ng/mL) the lower chamber at a concentration determined in preliminary experiments to induce IL2. Add 100 µL of the cell suspension to the upper chamber. Incubate the chamber at 37°C with 5% CO_2_ for 2–4 h. After incubation, remove the upper chamber and gently wash the lower chamber with PBS. Detach the migrated cells using trypsin or by scraping. Count the cells using a hemocytometer. In separate experiments, same Treg cells were pretreated with different drugs, including Rapamycin (an mTOR inhibitor), 2‐DG (a glycolysis inhibitor), and Metformin (a glycolysis stimulator) to assess their migration towards CXCL11 (200 ng/mL).

### 4.4 Treg cell recruitment from circulation to peritoneal cavity

Labelled Tregs (1 × 10^6^ cells/mL /mouse) were injected i.v. T cells were incubated at 37°C for 30 min and then washed three times with PBS before injection. After 24 h, mice were sacrificed, tissues were sampled and either processed for flow cytometric analysis (lymph nodes and spleen) or embedded in optimal cutting temperature compound (Agar Scientific, Stansted, UK), snap‐frozen and stored until analysis. Tissue infiltration by T cells was assessed by wide‐field fluorescence microscopy.

### 4.5 Extracellular acidification rate and oxygen consumption rate measurement in Seahorse Analyzer

ECAR and OCR measurements were conducted using a Seahorse Analyzer to assess cellular metabolism in CD4+ T cells, including WT, KO, and mutant cells. Cells (6 × 10^5^/well) were seeded in a Seahorse XF plate and allowed to adhere overnight. Prior to the assay, the culture medium was replaced with a non‐buffered solution to minimise interference. The plate was then placed in the Seahorse Analyzer, where baseline measurements of ECAR and OCR were recorded. Following baseline measurements, specific metabolic inhibitors and substrates (glucose, oligomycin and 2DG for ECAR; oligomycin, FCCP, rotenone/antimycin A for OCR) were sequentially injected into the wells to evaluate glycolytic and mitochondrial function. After each injection, the analyzer measured the changes in ECAR and OCR, allowing for the calculation of key metabolic parameters. Data were analysed to determine the metabolic profile of the cells under different conditions.

### 4.6 Glucose uptake assay

Cells were cultured in RPMI‐1640, supplemented with 10% fetal bovine serum (FBS), 1% penicillin–streptomycin and allowed to reach 70%–80% confluence. The media was replaced with glucose‐free medium and incubated for 1 h to starve the cells. Subsequently, 6‐NBDG (a fluorescent glucose analog) was added to each well at a final concentration of 100 µM, and the cells were incubated for 30 min at 37°C. After incubation, the cells were washed twice with PBS to remove excess 6‐NBDG. Fluorescence was measured using a fluorescence microplate reader with an excitation wavelength of 485 nm and an emission wavelength of 530 nm. The amount of glucose uptake was quantified by comparing the fluorescence intensity to a standard curve generated with known concentrations of 6‐NBDG. Results were expressed as relative fluorescence units (RFU) per milligram of protein, normalised against control conditions. All assays were performed in triplicate to ensure reproducibility.

### 4.7 Stable isotope tracer analysis

Stable isotope tracer analysis was conducted as previously described.^34^ Cells were cultured in glucose‐free DMEM (cat. no. D6429, Sigma‐Aldrich) supplemented with 12 mM 13C6‐fructose (cat. no. 100145, Cambridge Isotope Laboratories, Andover, MA) or 13C6‐glucose (cat. no. 100144, Cambridge Isotope Laboratories) and 10% dialysed FBS (cat. no. 26400, Thermo Fisher Scientific) for 12 h. Following collection and washing with cold PBS, cell pellets were extracted with 60 µL methanol (cat. no. 32221, Thermo Fisher) and homogenised using a Bullet Blender (Next Advance, Troy, NY) for 3 min. The supernatant was derivatised with 20 µL of a freshly prepared reagent (200 mM 3‐NPH in 75% methanol and 96 mM EDC‐6% pyridine) at 30°C for 60 min. Samples were then lyophilised, dissolved in 200 µL of ice‐cold 50% methanol, and analysed via UPLC‐QTOF‐MS (Waters Corp., Milford, MA) using an Acquity BEH C18 column (100 mm × 2.1 mm, 1.7 µm).

### 4.8 ATP consumption assay

Cells were cultured in 2 mL of RPMI 1640 medium with 25 mM glucose (cat. no. G8270, Sigma‐Aldrich, St. Louis, MO) or 25 mM fructose (cat. no. F0127, Sigma‐Aldrich). Following a 24‐h incubation, ATP levels were quantified using the ATP Assay Kit (cat. no. A22066, Thermo Fisher Scientific, Waltham, MA). A 100 µL sample of cell supernatant was mixed with 100 µL of ATP detection reagent from the kit. Luminescence was measured using a luminometer.

### 4.9 Pull‐down assay

Cell lysates were prepared from cultured cells, and an appropriate amount of lysate was incubated with a specific bait protein immobilised on beads. The mixture was rotated at 4°C for several hours to allow binding. After incubation, the beads were washed multiple times with a wash buffer to remove unbound proteins. The bound proteins were eluted from the beads using an elution buffer. The eluted samples were analysed by SDS‐PAGE followed by Western blotting to detect the presence of the target proteins. Band intensity was quantified using imaging software to evaluate the interaction strength.

### 4.10 Western blotting and densitometry analysis

The following primary antibodies were used in western blotting: CD31+ (catalog no. SAB5700639), FOXP3+ (catalog no. SAB5300461), β‐actin (catalog no. A5441), P‐tyrosine (catalog no. SAB5600274), and SHP2 (catalog no. SAB5701201). Meanwhile, from Invitrogen, the antibodies used were RNF111 Polyclonal Antibody (catalog no. PA5‐106655), PFKFB3 (catalog no. MA5‐32766), PAk4 (catalog no. MA5‐268590), cald1 (catalog no. MA5‐56509), Arhgdib (catalog no. MA1‐41085), Rac1 (catalog no. PA1‐091), Rock1 (catalog no. PA5‐22262), Nck1 (catalog no. MA5‐15024), Cdk5r1 (catalog no. MA5‐14834), Arap1 (catalog no. MA1‐19728), RAS (catalog no. 33–7200), p‐ERK (catalog no. 44–680G), ERK (catalog no. MA5‐15134) and RAC (catalog no. 44–609G). These antibodies were critical for detecting and analysing the presence and activity of these proteins in various experimental conditions.

Densitometry analysis was performed to quantify protein bands from Western blot images. The blots were scanned at a consistent resolution to ensure uniformity. Using densitometry software, regions of interest corresponding to the protein bands were selected. The software calculated the optical density of each band, allowing for comparison between samples. Background noise was subtracted to enhance accuracy, and the intensity of the target bands was normalised to a loading control to account for variations in protein loading. Data were analysed to determine relative protein expression levels across different samples.

### 4.11 Liquid chromatography–mass spectrometry identification

10^7^ WT/CD31 KO Tregs ± 100 nM CXCL11 were collected and homogenised with a mixture of Millipore ultrapure water and cold methanol. The metabolites were assayed utilising a Waters ACQUITY UPLC system along with UV detector set at 254 nm. The chromatographic separation was achieved on Waters BEH C18 Column 1.7 µm, 2.1 mm×100 mm. Mobile phase component A was water with 7 mM TEA and 100 mM HFIP. Mobile phase component B was methanol. The metabolites were separated using gradient elute (.5 min, 5% phase B;5 min, 100% phase B), accompanied by the flow rate.2 mL/min.

### 4.12 Pathway‐focused RT‐PCR array

Employ the RT‐PCR array system (Cell Motility RT2 Profiler PCR Array; Qiagen) as previously described^21^ to evaluate the activity of key signalling pathways. RNAs were isolated and converted to cDNA using the RT2 First Strand Kit. Then aliquot this mixture (25 µL for 96‐well or 10 µL for 384‐well plates) to each well of the same RT2 Profiler PCR Array plate containing the predispensed gene‐specific primer sets and perform PCR. Use 7500 Fast System SDS Software (Applied Biosystems) to calculate the threshold cycle (CT) values for all the genes on each RT2 Profiler PCR Array. Finally, calculate fold changes in gene expression for pairwise comparison using the ∆∆CT method.

### 4.13 STRING database analysis and pathway enrichment

The STRING database is a well‐established tool specifically designed for obtaining information about protein–protein interactions and a comprehensive dataset that includes a significant number of experimentally validated interactions. A list of genes (DEGs) was selected from CD31‐related pathways based on results from Cell Motility RT2 Profiler PCR Array. We then performed the KEGG pathway enrichment analysis and GO Biological Pathway analysis on website: http://bioinformatics.sdstate.edu/go/. The details are covered in refs.^35^, ^36^, ^37^ We also performed protein–protein interaction analysis on STRING Database website: https://string‐db.org/cgi/input?sessionId = bQ6ZvYjt7l6e&input_page_show_search = on, which is covered in ref.,^38^ using the gene sets in the article.^39^

### 4.14 Immunofluorescence staining

Immunofluorescence staining in cultured cells was performed as follows: Cells were gently rinsed twice with cold PBS and then fixed for 40 min in a 4% formaldehyde solution (pH 7.4) at room temperature (RT). The fixed cells were then permeabilised in a 0.1% Triton X‐100 solution on ice for 10 min and incubated overnight at 4°C with primary antibodies against DAPI (Sigma‐Aldrich, D9542), FOXP3+ (Sigma‐Aldrich, SAB5300461), CD45+ (Sigma‐Aldrich, SAB4502541), CD68+ (Invitrogen, 14‐0681‐82), GR1+ (Cell Signalling, 31469), SHP2 (Sigma‐Aldrich, SAB5700078), CD31 (Sigma‐Aldrich, SAB5700639), RNF111 (Sigma‐Aldrich, HPA038576), CD8+ (Thermo Fisher, 740029TP555), OGT (Sigma‐Aldrich, O6014), and CD4+ (Sigma‐Aldrich, SAB4503583). This was followed by staining with the secondary antibody which had been linked to Alexa Fluor 594 (A‐11032, Invitrogen). The cells were then stained with Hoechst 33342. Images were obtained using laser confocal microscopy (Leica Microsystems).

### 4.15 Immunohistochemistry

IHC was performed as follows: after dewaxing and rehydration, endogenous peroxidase was eliminated by treatment with 3% hydrogen peroxide. The slides were then incubated at 4°C overnight with primary antibodies: FOXP3+ (Sigma‐Aldrich, SAB5300461), CD4+ (Sigma‐Aldrich, SAB4503583), CD25+ (Sigma‐Aldrich, MABF2101), TNF alpha (Sigma‐Aldrich, SAB5700698), and IFN gamma (Sigma‐Aldrich, I5027). The staining scores were calculated according to the proportion of the staining‐positive area in 10 random fields under a 40× objective. The percentage of positive staining of tumour cells on the slides was scored as follows: 0, no positive stained area; 1, less than 25%; 2, 25%–50%; and 3, more than 50%. The exhibited staining score was the total number of the 10 random fields.

### 4.16 Haematoxylin and Eosin staining

Tissue sections were initially deparaffinised in xylene and rehydrated through a series of graded ethanol solutions. The sections were then immersed in haematoxylin for approximately 5–10 min, followed by rinsing in running tap water to remove excess stain. After that, the sections were differentiated in a solution of hydrochloric acid in alcohol and subsequently rinsed again. Eosin was applied for about 1–2 min to stain the cytoplasm, and the sections were washed in water to remove unbound dye. Finally, the sections were dehydrated through graded ethanol, cleared in xylene, and mounted with a coverslip using a suitable mounting medium. Stained sections were examined under a microscope to assess tissue architecture and cellular details.

### 4.17 Cellular internalization assay by confocal imaging

Tregs cells were seeded in glass‐bottom confocal dishes at a density of 5 × 10^4^ cells per well and cultured overnight. The following day, cells were treated with 250 nM of 5′‐Cy5‐labelled aptamer‐siRNA. Alexa Fluor 488‐labelled endocytic markers (50 µg/mL dextran, 50 µg/mL transferrin and 5 µg/mL CTX‐B) were added into the cells and incubated at 37°C for 3 h. After incubation, cells were washed three times with PBS. 1 mL of 4% PFA was added to each well incubated for 10 min at RT to fix cells. The cells were washed three times with PBS, and stained with 4 µg/mL DAPI for 10 min. The fixed and stained cells were washed and visualised using Leica SP5 X laser scanning confocal microscope. Images were acquired with the spectral detector of the microscope using appropriate emission wavelength ranges; DAPI (blue) was excited at 352 nm using the argon laser, with emission recorded between 440 and 460 nm. Cy5 was excited at 649 nm with the DPSS laser, and emission was recorded between 660 and 680 nm. Alexa Fluor 488 (green) was excited at 480 nm with the DPSS laser, and emission was recorded between 510 and 530 nm. Images were acquired using the LAS X software (Leica Microsystems CMS GmbH, Wetzlar, Germany) and presented without any post‐processing. Photos were either taken with an Axioplan2 or a LSM510 (confocal), both from Zeiss with a 60× objective.

### 4.18 Flow cytometry analysis

Cell samples are harvested and suspended in FACS buffer (PBS, 1% BSA, 0.01% sodium azide), fixed using fixation buffer (PBS, 4% paraformaldehyde, 1% FCS), to remove any residual culture medium. For surface marker staining, cells were resuspended in 100 µL of staining buffer (PBS with 2% FBS) and incubated with appropriate concentration of fluorescence‐conjugated antibodies, or isotype control antibodies at the appropriate dilution as recommended by the manufacturer. The samples were incubated for 30 min at 4°C in the dark to prevent photobleaching. Following incubation, cells were washed twice with staining buffer to remove unbound antibodies. For intracellular staining, cells were fixed and permeabilised according to the manufacturer's protocol. Cells were then stained with PKH26+(Sigma‐Aldrich, MIDI26); P‐tyrosine (Sigma‐Aldrich, SAB5600274); CD25(Sigma‐Aldrich, MABF2101); 6 NBDG fluorescence, (Sigma‐Aldrich, 72987); SHP2, (Sigma‐Aldrich, ZRB1934); OGT Polyclonal Antibody (Invitrogen, PA5‐22071); anti‐CD4 (Mouse) (Sigma‐Aldrich, MABF157B); Monoclonal Anti‐FOXP3‐PE antibody (Sigma‐Aldrich, SAB4700611) for 30 min at 4°C in the dark. After staining, cells were washed twice with permeabilisation buffer.

Flow cytometric analysis was performed using a BD 831 Immunocytometry Systems. Data were acquired using FlowJo 7.6 software (Tree Star, Inc.) and analysed to determine the expression levels of the targeted markers and statistical analysis was conducted using SPSS 16.0. All experiments were performed in triplicate to ensure reproducibility.

### 4.19 Lactate and glucose measurement

Cells were cultured in DMEDM (Gibco, 11965092) with 10% FBS (Gibco, 16000044) until confluent. Media was replaced with fresh medium containing glucose (5–25 mM; Sigma, G7021), fructose (5 mM; Sigma, F0127), with or without 2DG (10 mM; Sigma, D8375). After 24 h, supernatants were collected. Lactate levels were quantified using the Lactate Assay Kit II (BioVision, K627‐100): 50 µL supernatant mixed with 50 µL assay reagent, incubated for 30 min at 37°C, and measured at 450 nm (microplate reader, BioTek Synergy H1). Glucose concentrations were determined via the Glucose Colorimetric Assay Kit (Sigma, MAK263): 10 µL supernatant + 90 µL assay reagent, incubated for 30 min at 37°C, and read at 540 nm.

### 4.20 Knockdown experiments using siRNA

PFKFB3 was silenced using ON‐TARGET plus Human PFKFB3 siRNA (Dharmacon, L‐006309‐00‐0005) at 50 nM. Cells were seeded in 6‐well plates (3×10⁶ cells/well) in DMEM (Gibco, 11965092). For transfection, 5 µL siRNA (stock: 20 µM) was diluted in 245 µL Opti‐MEM (Gibco, 31985070) and mixed with 7.5 µL Lipofectamine RNAiMAX (Invitrogen, 13778150) diluted in 242.5 µL Opti‐MEM. After 5 min incubation, the siRNA‐lipid complex (500 µL total) was added to cells (final siRNA: 50 nM). Cells were harvested 24 h post‐transfection. Knockdown efficiency was validated via Western blot (anti‐PFKFB3 antibody: Abcam, ab181861, 1:1000). Scrambled siRNA (Dharmacon, D‐001810‐10‐05) served as control. All steps followed manufacturer protocols.

### 4.21 Statistical analysis

The results are expressed as means ± SD. Each experiment was repeated at least three times. Significant differences between multiple groups were identified by one‐way ANOVA. Whenever two groups were compared, Student unpaired *t*‐tests were performed. All reported *P*‐values are two sided. A *P*‐value < 0.05 was regarded as significant.

## AUTHOR CONTRIBUTIONS

**Meijun Liu, Wengqiong Huang and Xiaoli Chen**: Writing—review & editing, Writing—original draft, investigation and conceptualization. **Zongzhen Meng, Jiawen Yang, Rodrigo Azevedo, Xiaojiao Zheng and Hao Shen**: Visualization and data curation. **Wei Jia and Aiping Lyu**: Investigation. **Kenneth Chat Pan Cheung**: Supervision, project administration, investigation, conceptualization.

## CONFLICT OF INTEREST STATEMENT

The authors declare no financial or commercial conflicts of interest.

## ETHICS STATEMENT

The study was approved by the Ethics Committee of Shanghai Sixth People's Hospital and Ethics Committee on Laboratory Animals of Hong Kong Baptist University (REC/20‐21/0584). All participants provided written informed consent.

## Supporting information



Supporting information

Supporting information

Supporting information

## Data Availability

All data generated or analysed during this study are included in this article. Further enquiries can be directed to the corresponding author upon reasonable request.
