# Supplementary figures and images for: CD31 regulates metabolic switch in Treg migration attenuates rheumatoid arthritis
Source: Clin Transl Med. 2025 Aug 15;15(8):e70441. doi: 10.1002/ctm2.70441 (PMC12356826; doi:10.1002/ctm2.70441)

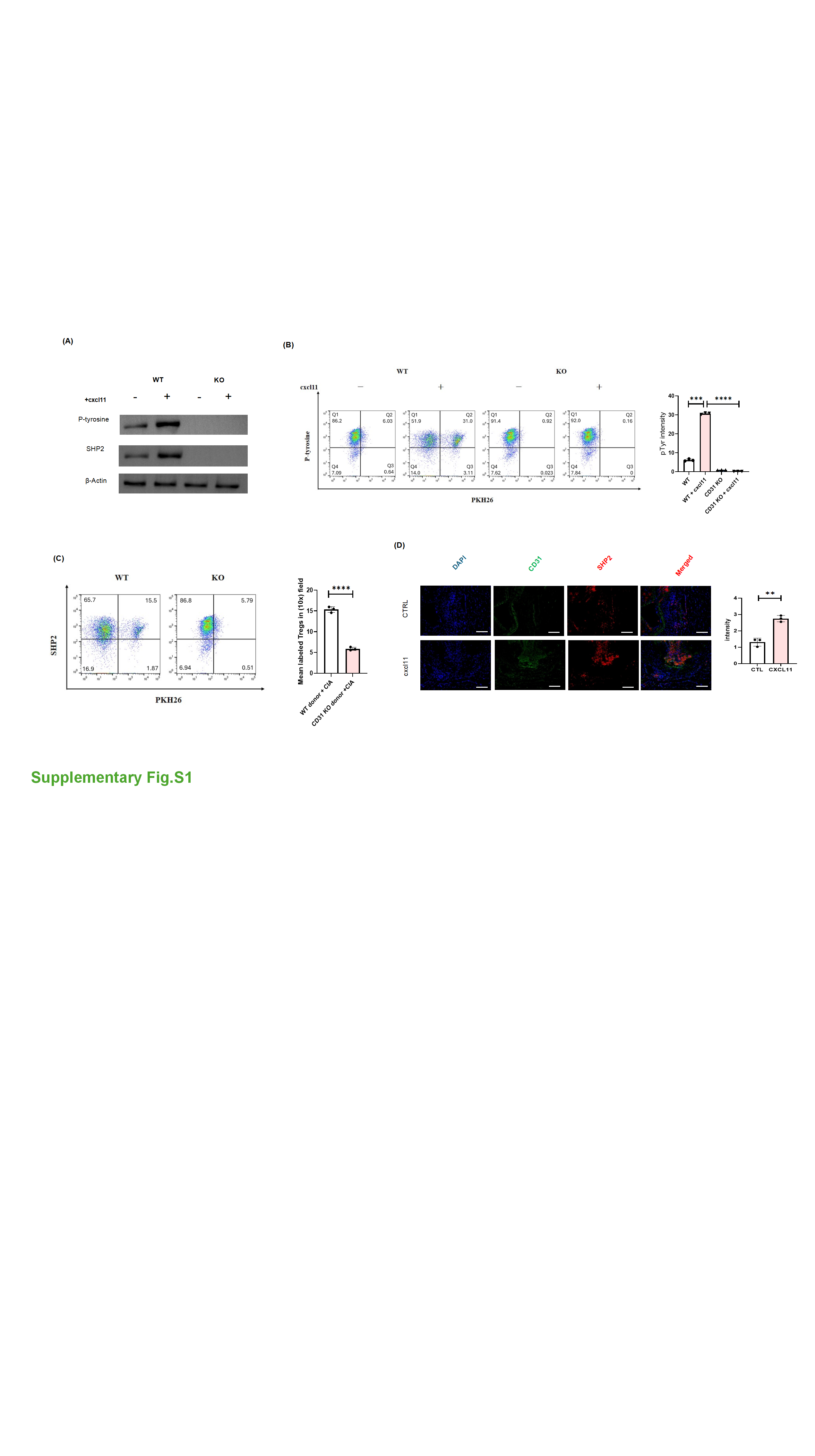

Supplement: Supplementary file 1 — Supporting information [file CTM2-15-e70441-s002.tif]

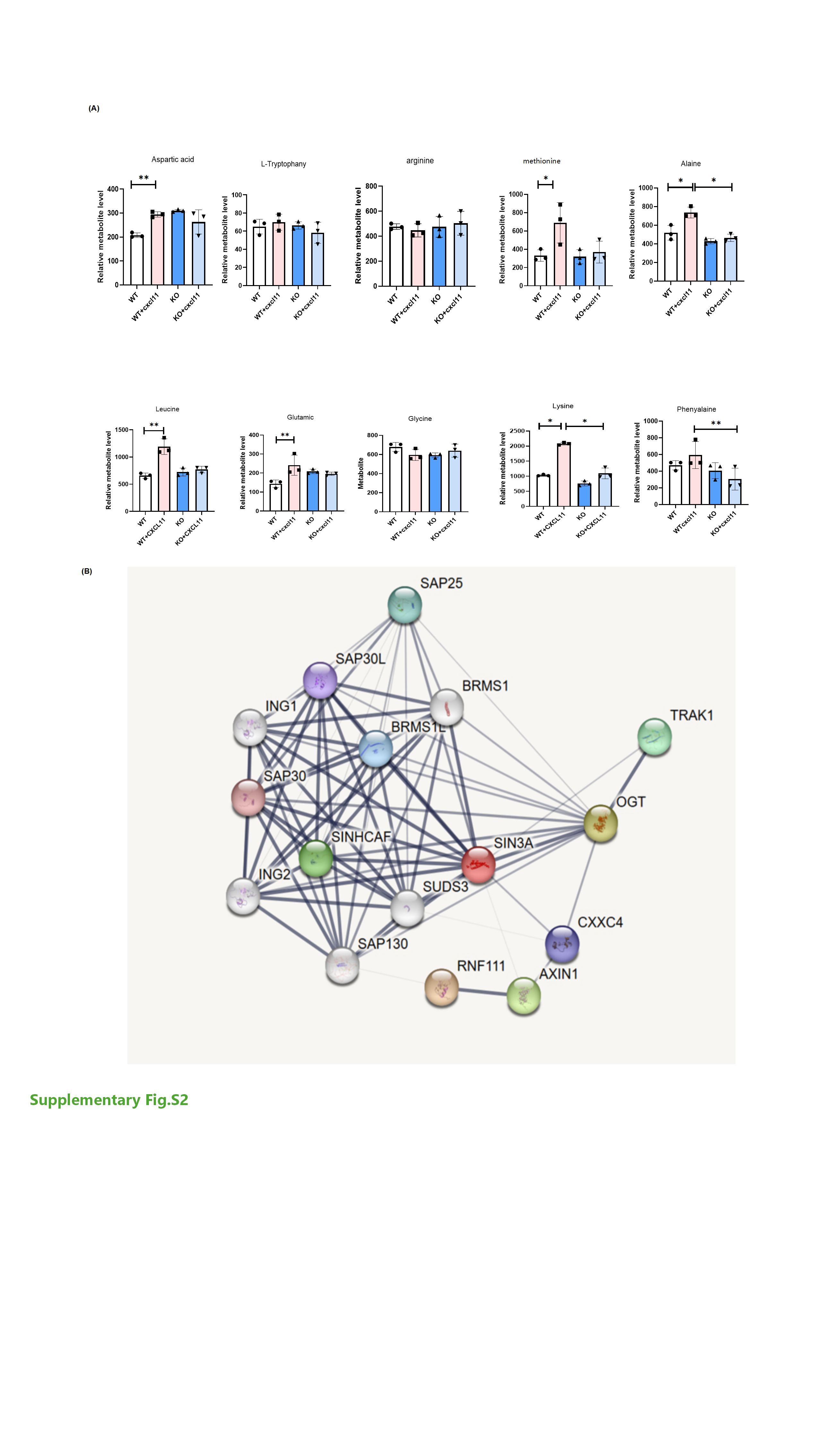

Supplement: Supplementary file 2 — Supporting information [file CTM2-15-e70441-s001.tif]

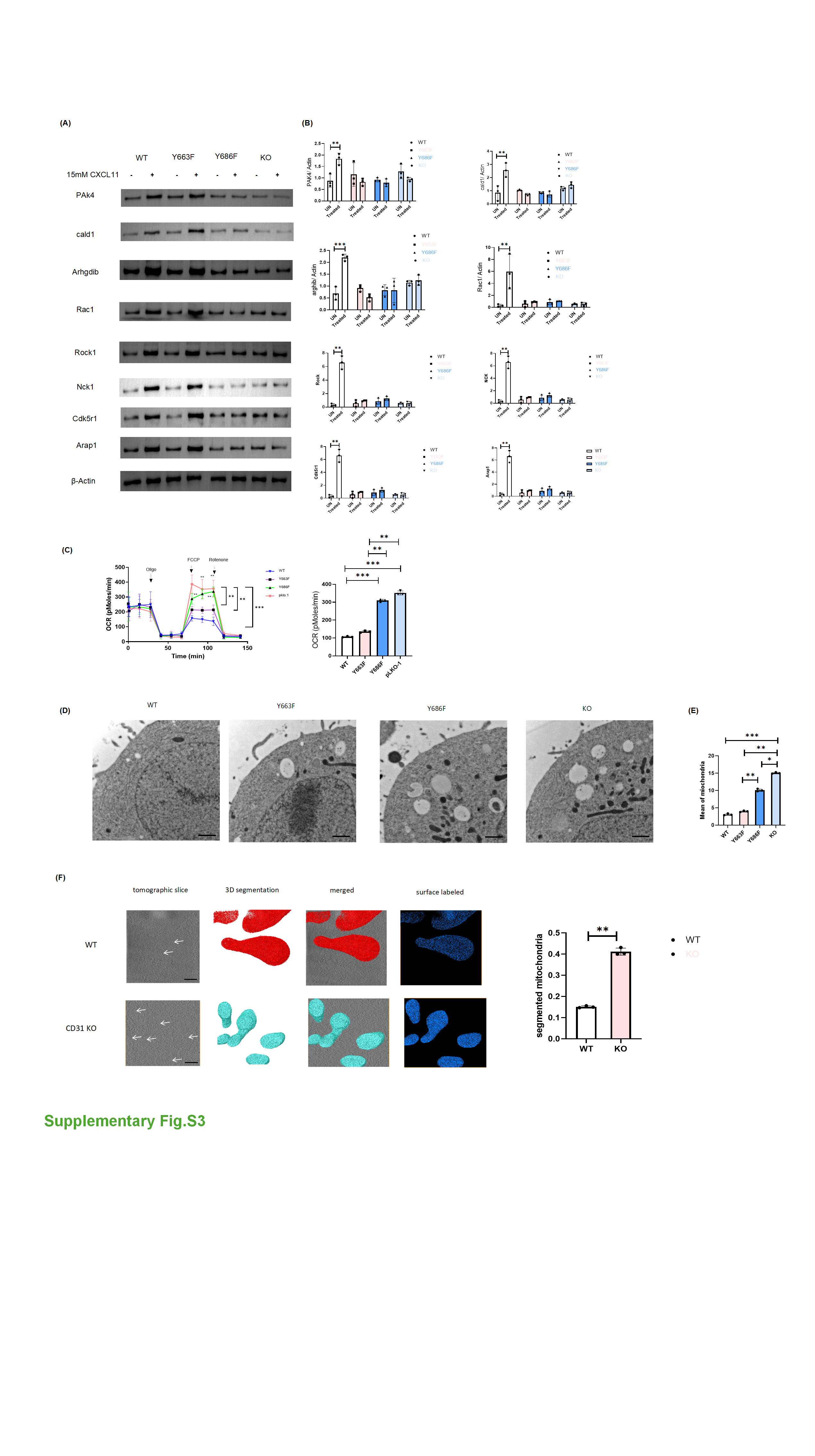

Supplement: Supplementary file 3 — Supporting information [file CTM2-15-e70441-s003.tif]
